# Supplementary material for: The MpsAB Bicarbonate Transporter Is Superior to Carbonic Anhydrase in Biofilm-Forming Bacteria with Limited CO2 Diffusion
Source: Microbiol Spectr. 2021 Jul 21;9(1):10.1128/spectrum.00305-21. doi: 10.1128/spectrum.00305-21 (PMC8552792; doi:10.1128/spectrum.00305-21)
Supplement: SUPPLEMENTAL FILE 1 — Supplemental material. Download SPECTRUM00305-21_Supp_1_seq7.pdf, PDF file, 1.9 MB. [file spectrum00305-21_supp_1_seq7.pdf]

SUPPLEMENTARY MATERIALS

The MpsAB bicarbonate transporter is superior to carbonic anhydrase in biofilm-forming bacteria with limited CO_2 diffusion

Sook-Ha Fan,[a] Miki Matsuo,[a] Li Huang, [a,b] Paula M. Tribelli,[a,c] and Friedrich Götz[a #]

SUPPLEMENTARY FIGURES

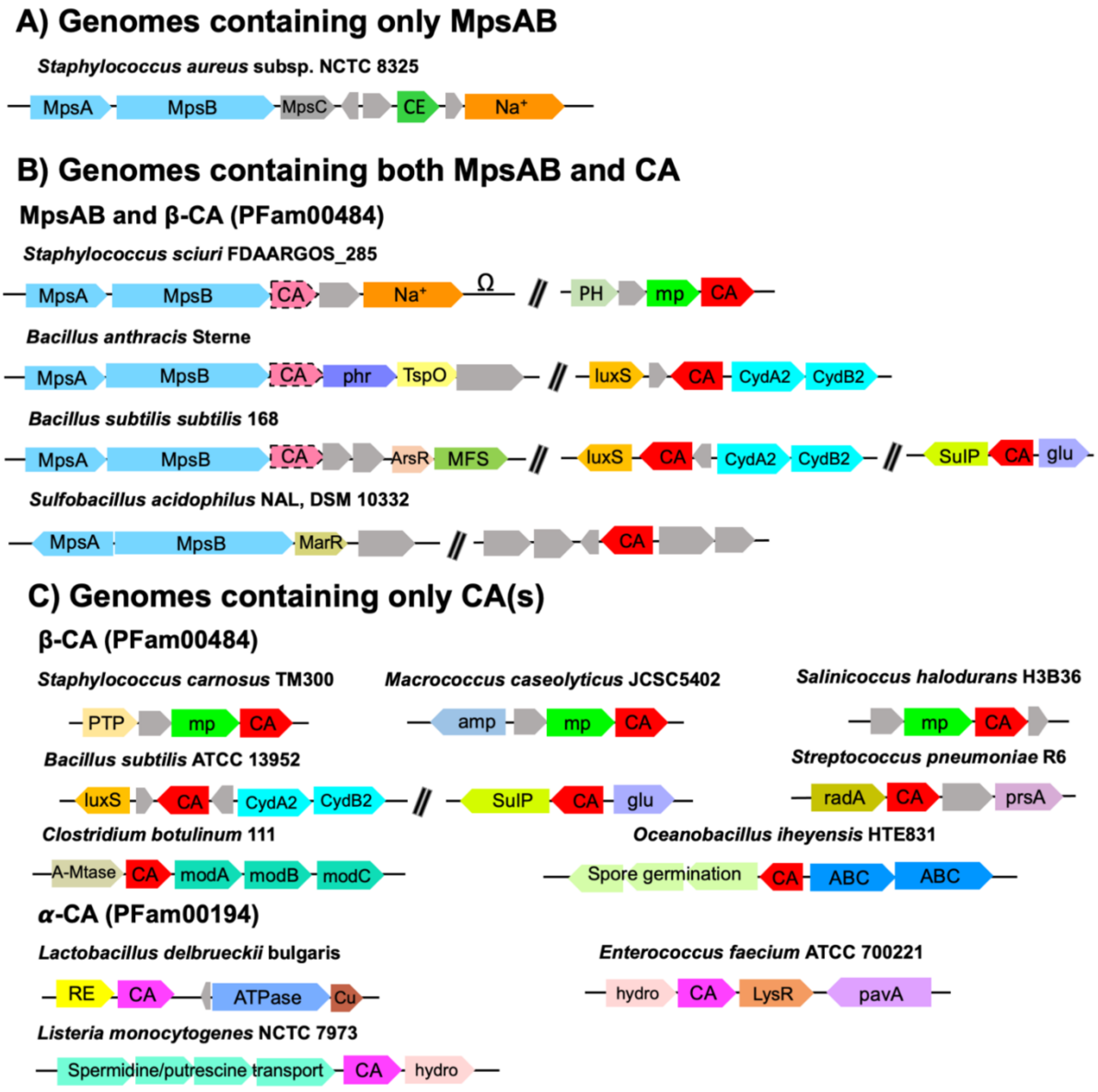

Supplementary Figure 1: Gene synteny of the genome organization around CA in selected *Firmicutes* genomes. Figures were constructed based on the CA PFam motif of selected Firmicutes genomes found in Integrated Microbial Genomes & Microbiomes (IMG/M) database (1). Gene synteny shows genomes containing **(A)** MpsAB only **(B)** both MpsAB and β-CA (s) while **(C)** shows genomes containing only CAs which can be further categorized by the presence of only β-CAs (PFam00484 for prokaryote CA), α-CAs (PFam00194 for eukaryote CA) or both. The terms β-CA

for PFam00484 and α-CA for PFam00194 are used in the figure in order to more adequately describe the evolutionary history of these enzymes. PFam00484 is colored in red whereas PFam00194 is colored in magenta. The dashed line lining the light red colored CAs in (A) are proteins annotated as putative CA but without the related pFam motif. Same color schemes are used for proteins of similar functions/families and/or if they belong to the same operon. Genes in grey indicate hypothetical proteins or uncharacterized proteins. The omega symbol (Ω) indicates a transcriptional terminator. For clarity, CA genes mostly point to the right, even if it is located on the complementary strand.

The following are the acronyms used:

A-Mtase, putative N6-adenine specific DNA methylase; ABC, ABC transporter ATP-binding protein, amp, leucyl aminopeptidase; ArsR, Arsenical resistance operon (ArsR) family transcriptional regulator; ATPase, cation transport ATPase; CA, carbonic anhydrase; CE, carboxylesterase; Cu, copper chaperone, CydA2, putative cytochrome bd-I ubiquinol oxidase subunit 1 apoprotein; CydB2, putative cytochrome bd-I ubiquinol oxidase subunit 2 apoprotein; glu, gluconate 2-dehydrogenase; hydro, putative hydrolases of HD family; isomerase, sugar phosphate isomerase/epimerase, luxS, S-ribosyl homocysteine lyase; LysR, DNA-binding transcriptional regulator, LysR family; MarR, Multiple antibiotic resistance Repressor family protein, MFS, Major Facilitator Superfamily; modA, molybdate transport system substrate-binding protein, modB, molybdate transport system permease protein; modC, molybdate transport system ATP-binding protein; mp, membrane protein; MpsA, Membrane potential-generating system A; MpsB, Membrane potential-generating system B; MpsC, Membrane potential-generating system C; Na+, phosphate sodium transporter; OmpR, DNA-binding response regulator, OmpR family; pavA, predicted

component of the ribosome quality control (RQC) family; spermidine/putrescine transport, spermidine/putrescine transport system substrate-binding protein, PH, Pleckstrin Homology (PH)-domain containing protein; phr, deoxyribodipyrimidine photo lyase type 1; prsA, ribose-phosphate pyrophosphokinase; PTP, Protein Tyrosine Phosphatase; radA, DNA repair protein; RE, restriction endonuclease, spore germination, spore germination protein; SulP, Sulfate Permease family; TspO, Tryptophan-rich sensory protein/Mitochondrial Benzodiazepine Receptor (TspO/MBR) related proteins.

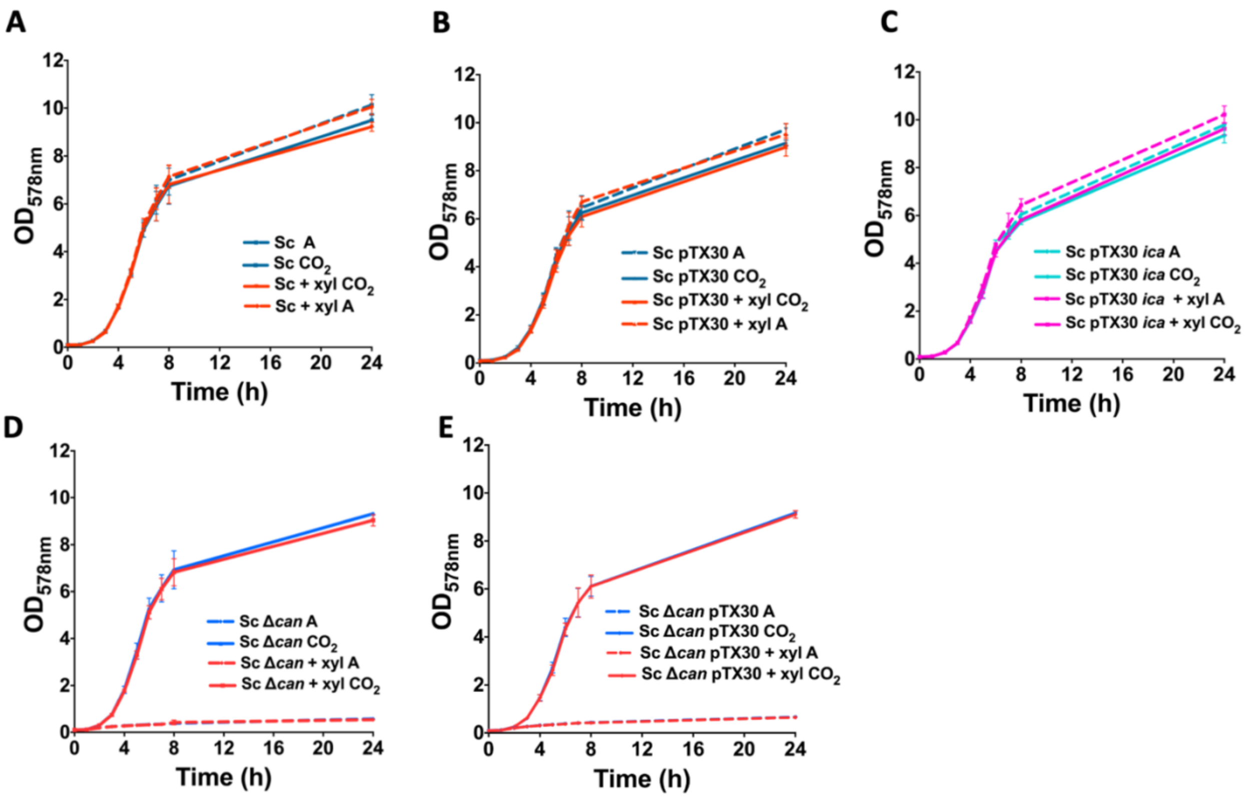

Supplementary Figure 2: **The growth of S. carnosus (Sc) TM300 and TM300 carbonic anhydrase deletion mutant (Δ*can*) were not affected by the addition of xylose (xyl) , empty plasmid pTX30 or biofilm in both atmospheric, A and CO_2 conditions.** **(A)** The growth of Sc TM300 wild type with and without the addition of xylose at a final concentration of 0.7%. **(B)** The growth of Sc TM300 carrying the empty plasmid pTX30 as control, with and without the addition of 0.7% xylose. **(C)** The growth of Sc TM300 carrying plasmid pTX30 with *ica* genes encoding biofilm associated genes with and without the addition of 0.7% xylose as inducer. **(D)** The growth of Sc Δ*can* with and without the addition of xylose at a final concentration of 0.7%. **(E)** The growth of Sc Δ*can* carrying the empty plasmid pTX30 as control, with and without the addition of 0.7% xylose. All growth studies were performed using TSB. Each point in the graph is the mean ± SD from three independent biological replicates.

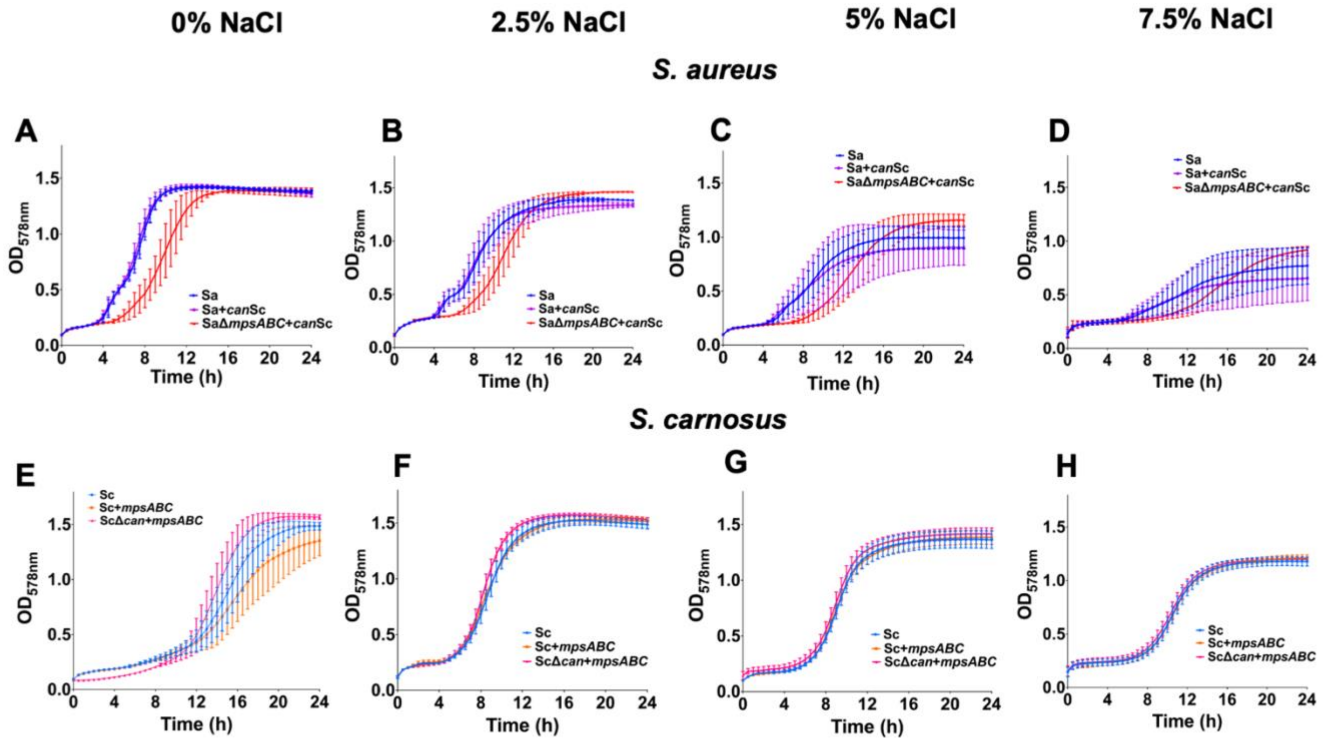

Supplementary Figure 3: **Growth of *S. aureus* (Sa) and *S. carnosus* (Sc) under salt stress tolerance.** Different concentrations of NaCl were added to LB media with *S. aureus* HG001 wild type (Sa), *S. aureus* HG001 with plasmid pRB473-*canSc* (*can* encoding CA from *S. carnosus)* (Sa+*can*Sc), and *S. aureus* HG001Δ*mpsABC* complemented with pRB473-*canSc* (SaΔ*mpsABC*+*can*Sc) **(A-D)** and also with *S. carnosus* TM300 wild type (Sc), *S. carnosus* TM300 with plasmid pRB473-*mpsABC* *(mpsABC* encoding 'bicarbonate transporter' from *S. aureus)* (Sc+*mpsABC*), and *S. carnosus*Δ*can* complemented with pRB473*mpsABC* (ScΔ*can*+*mpsABC*) **(E-H)** respectively. Concentrations used were **(A&E)** 0% Nacl **(B&F)** 2.5% NaCl **(C&G)** 5% NaCl and **(D&F)** 7.5% NaCl. Experiments were performed using microplate reader Varioskan Lux (Thermo Scientific) in a 48 wells plate. Each point in the graph is the mean ± SD from three independent biological replicates.

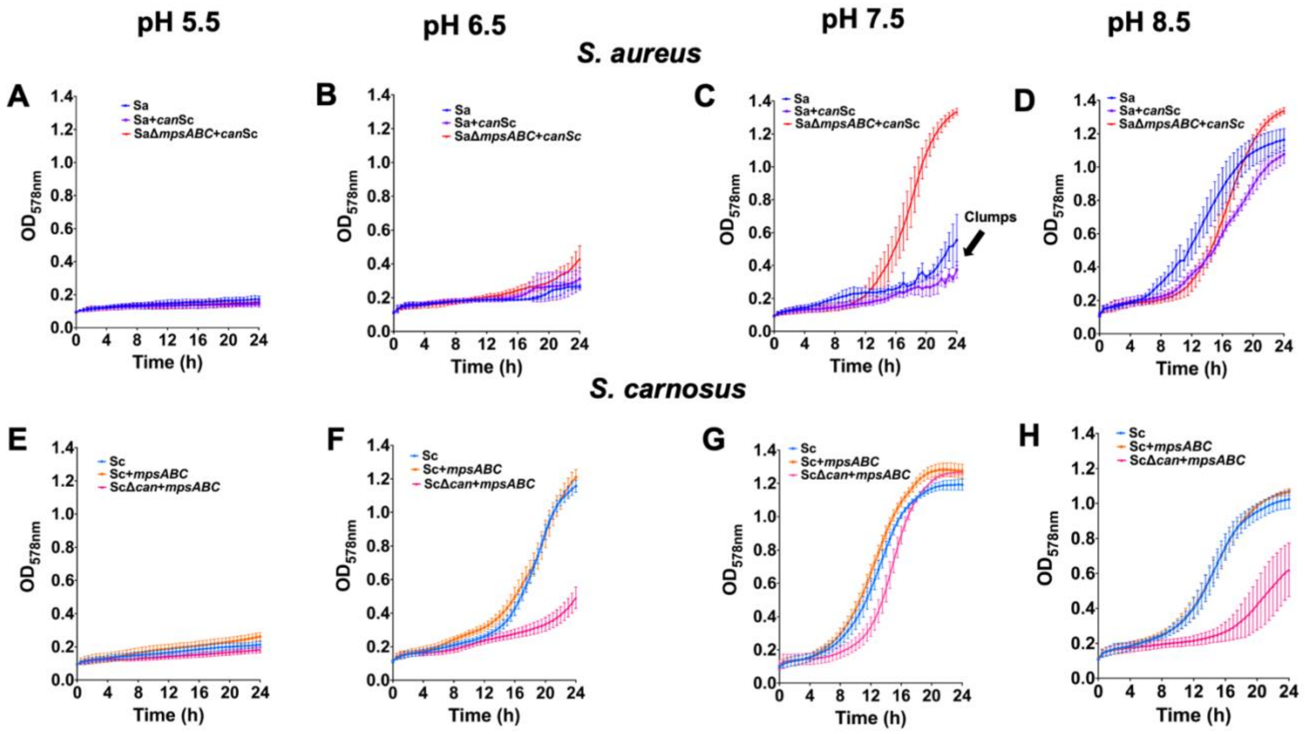

Supplementary Figure 4: Growth of *S. aureus* (Sa) and *S. carnosus* (Sc) under pH stress tolerance. LB media were fixed at different pH with *S. aureus* HG001 wild type (Sa), *S. aureus* HG001 with plasmid pRB473-*canSc (can* encoding CA from *S. carnosus)* (Sa+*can*Sc), and *S. aureus* HG001Δ*mpsABC* complemented with pRB473-*canSc* (SaΔ*mpsABC*+*can*Sc) **(A-D)** and also with *S. carnosus* TM300 wild type (Sc), *S. carnosus* TM300 with plasmid pRB473-*mpsABC (mpsABC* encoding 'bicarbonate transporter' from *S. aureus)* (Sc+*mpsABC*), and *S. carnosus*Δ*can* complemented with pRB473*mpsABC* (ScΔ*can*+*mpsABC*) **(E-H)** respectively. pH used were **(A&E)** pH 5.5 **(B&F)** pH 6.5 **(C&G)** pH 7.5 and **(D&H)** pH 8.5. Experiment was performed using microplate reader Varioskan Lux (Thermo Scientific) in a 48 wells plate. Arrow at **(C)** pH 7.5, clumps/cell agglutinations were seen in HG001 pRB473-*canSc and* HG001Δ*mpsABC* pRB473-*canSc,* leading to lower OD recorded by the microplate reader. Each point in the graph is the mean ± SD from three independent biological replicates.

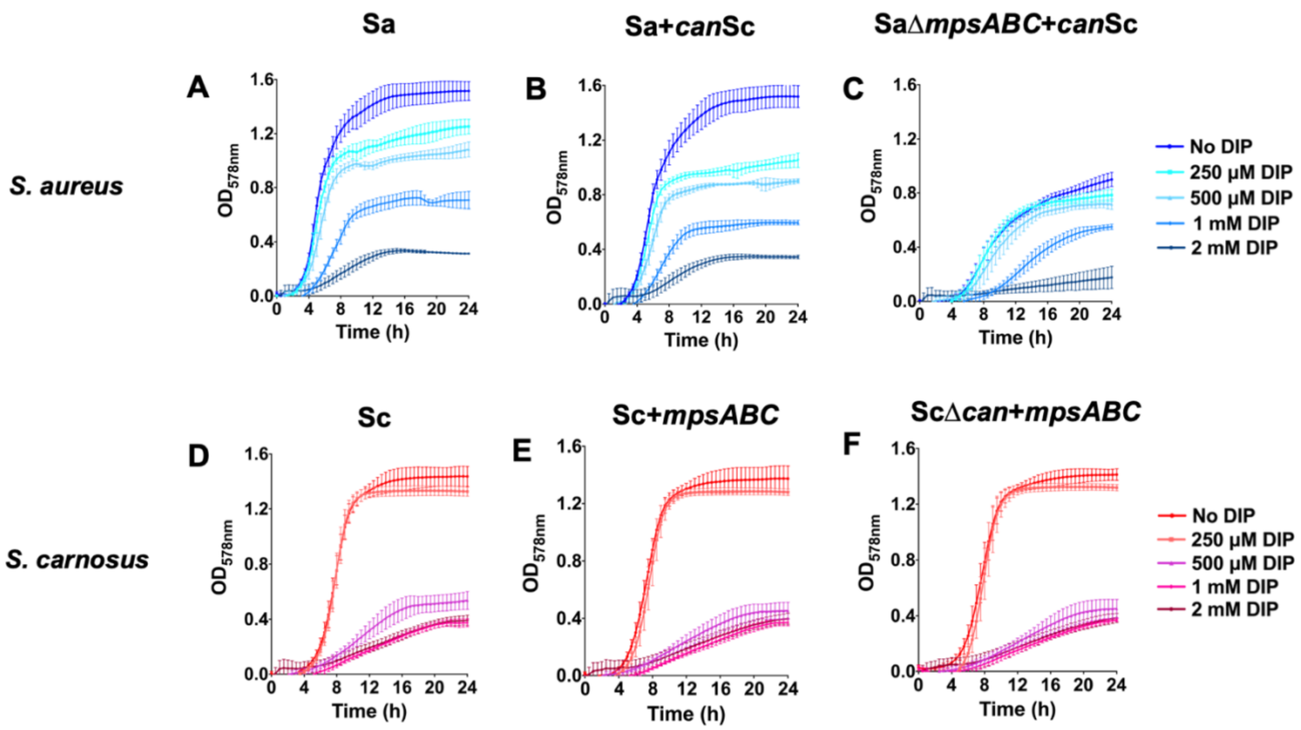

Supplementary Figure 5: Growth of *S. aureus* (Sa) (A-C) and *S. carnosus* (Sc) (D-F) under iron limitation conditions. Iron chelator 2,2 dipyridyl (DIP) at different final concentrations ranging from 250 µM to 2 mM were added to TSB media with **(A)** *S. aureus* HG001 wild type (Sa), **(B)** *S. aureus* with the addition of pRB473-*canSc* (Sa+*can*Sc), **(C)** *S. aureus* HG001Δ*mpsABC* complemented with pRB473-*canSc* (ScΔ*can+mpsABC*), **(D)** *S. carnosus* TM300 wild type (Sc), **(E)** *S. carnosus* with the addition of pRB473-*mpsABC* (Sc+*mpsABC*) and **(F)** *S. carnosus* TM300Δ*mpsABC* complemented with pRB473-*mpsABC* (ScΔ*can+mpsABC*). Experiments were performed using microplate reader Varioskan Lux (Thermo Scientific) in a 48 wells plate. Each point in the graph is the mean ± SD from three independent biological replicates.

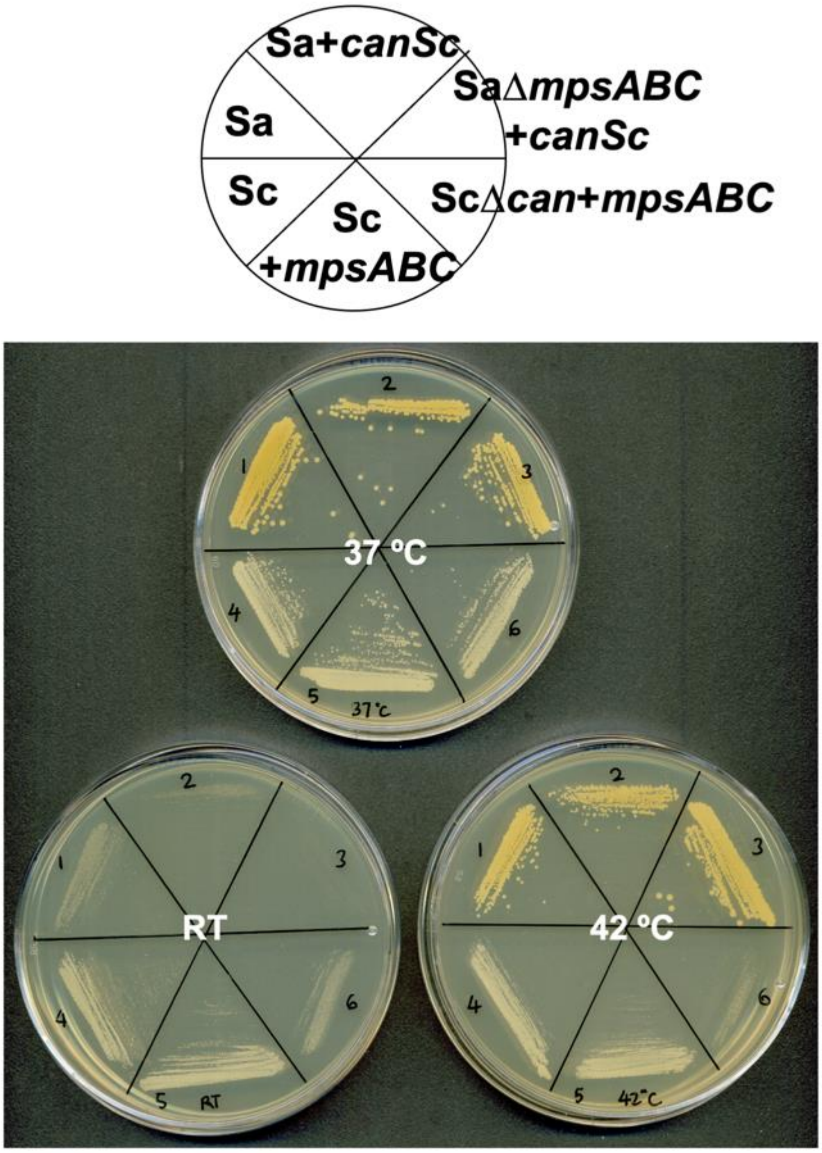

Supplementary Figure 6: **Growth of bacteria under temperature stress.** The strains used were as shown in the diagram, clockwise from the top: *S. aureus* HG001 with pRB473-*can*Sc(Sa+*can*Sc); *S. aureus* HG001Δ*mpsABC* complemented with pRB473-*can*Sc (SaΔ*mpsABC*+*can*Sc) ; *S. carnosus* TM300Δ*can* complemented with pRB473-*mpsABC* (ScΔ*can*+*mpsABC*); *S. carnosus* TM300 with the pRB473-mpsABC (Sc+*mpsABC*); *S. carnosus* TM300 wild type (Sc) and *S. aureus* HG001 wild type (Sa). Each strain was adjusted to OD_{578} of 0.5 and the inoculum was streaked on LB agar plate prior to incubation at room temperature (RT), 37 ºC and 42 ºC. Image of the plates were taken after 48 h incubation.

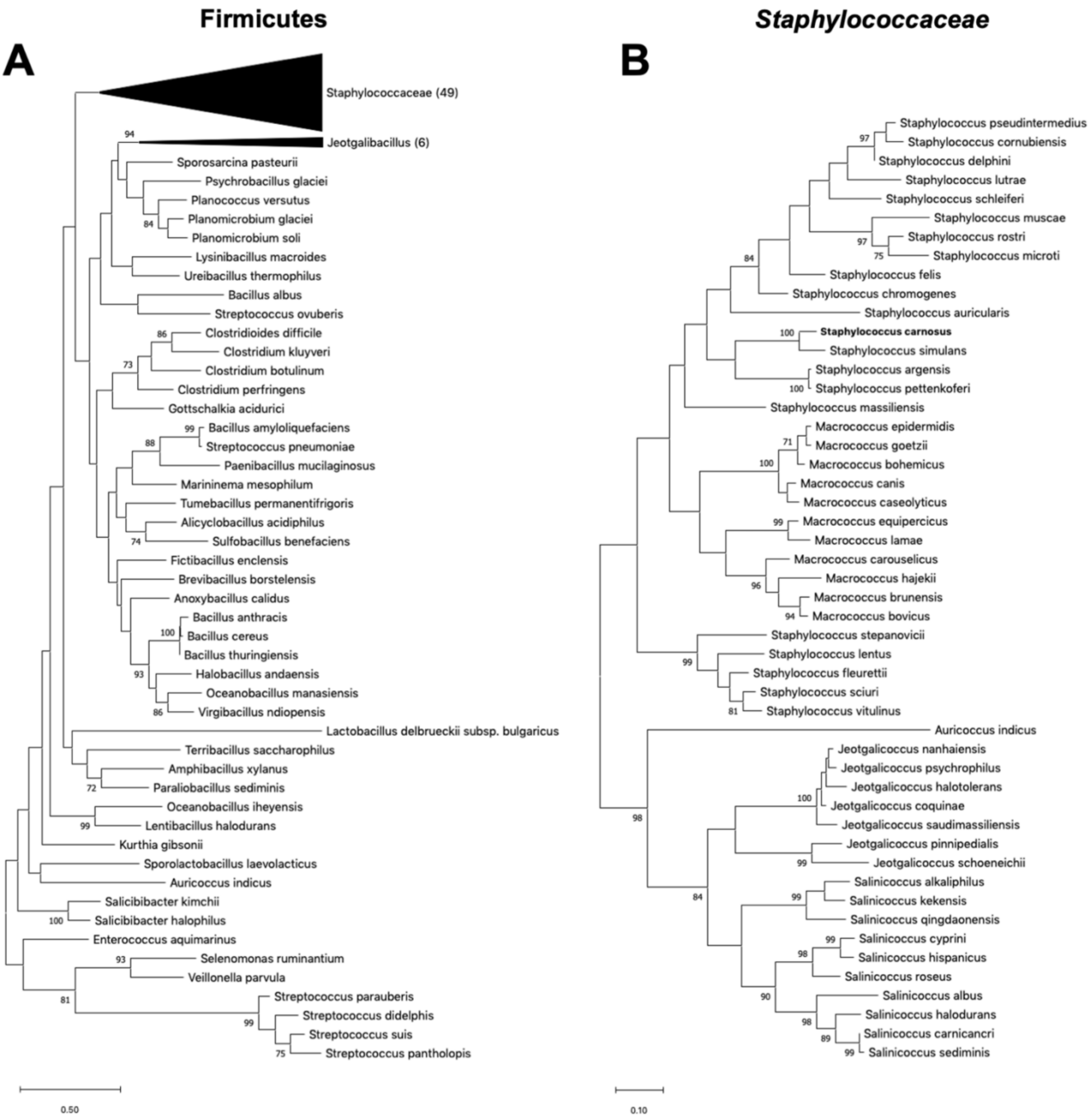

Supplementary Figure 7: Phylogenetic tree of CA in *Firmicutes* species.

Maximum likelihood phylogenetic analysis of the CA homologs among the selected species in **(A)** phylum *Firmicutes* and **(B)** family *Staphylococcaceae*. The CA protein involved in this study, *S. carnosus* is highlighted in bold. Node support is indicated by bootstrap values from 500 resampling of the alignment when they exceeded 70%. Collapsed clades are labeled by the shared taxonomic rank of the associated taxa. The number of species belonging to the respective clade is indicated in brackets.

SUPPLEMENTARY TABLES

Supplementary Table 1: The presence of MpsAB and/or CA in *Firmicutes* species

**Species**	**MpsAB**	**CA**	
		**β-CA (PFam 00484)**	**α-CA (PFam 00194)**
Acetobacterium woodii WB1, DSM 1030	-	+	-
Aeribacillus pallidus KCTC3564	+	-	-
Aerococcus christensenii CCUG28831	-	-	-
Aerococcus sanguinicola CCUG43001	-	+	-
Aerococcus urinae CCUG 36881	-	-	-
Aerococcus urinaeequi CCUG28094	-	+	-
Aerococcus viridans CCUG4311	-	+	-
Alicyclobacillus acidocaldarius acidocaldarius 104-IA, DSM 446	+	-	-
Alicyclobacillus acidocaldarius acidocaldarius Tc-4-1	+ *	-	-
Amphibacillus xylanus NBRC 15112	-	+	-
Anaerostipes hadrus BPB5	-	+	-
Anaerotignum propionicum X2, DSM 1682	-	+	-
Andreesenia angusta MK-1	-	++	-
Aneurinibacillus sp. XH2	-	+	-
Anoxybacillus sp. B2M1	+	-	-

Auricoccus indicus S31	-	+	-
Bacillus albus PFYN01	+	+	-
Bacillus altitudinis GQYP101	-	++	-
Bacillus amyloliquefaciens ALB69	-	++	-
Bacillus anthracis A16R	+ *	+	-
Bacillus anthracis Ames	+	+	-
Bacillus anthracis BF1	-	+	-
Bacillus anthracis Sterne	+	+	-
Bacillus aquimaris SAMM	-	+	-
Bacillus atrophaeus 1942	+	++	-
Bacillus beveridgei MLTeJB	-	+	-
Bacillus bombysepticus Wang	+	++	-
Bacillus cellulasensis GLB197	-	++	-
Bacillus cellulosilyticus N-4, DSM 2522	-	+	-
Bacillus cereus 03BB102	+	+	-
Bacillus clausii DSM 8716	-	+	+
Bacillus coagulans 2-6	-	+	-
Bacillus cohnii DSM 6307	-	+++	-
Bacillus cytotoxicus CH_13	+	+	-
Bacillus endophyticus Hbe603	-	++++	-
Bacillus flexus KLBMP 4941	-	+	-
Bacillus gibsonii FJAT-10019	+	++	-
Bacillus glycinifermentans B-27	+	++	-
Bacillus gobiensis FJAT-4402	-	++++	-

Bacillus halodurans C-125	-	+	+
Bacillus horikoshii 20a	-	+	-
Bacillus infantis NRRL B-14911	-	+	-
Bacillus kochii BDGP4	-	+	-
Bacillus krulwichiae NBRC 102362	-	++	-
Bacillus lehensis G1	-	+	+
Bacillus lentus NCTC 4824	-	+	+
Bacillus licheniformis ATCC 12713	+	++	-
Bacillus licheniformis MCC 2514	+ *	++	-
Bacillus megaterium DSM 319	-	++	-
Bacillus methanolicus MGA3	-	+++	-
Bacillus methanolicus PB1	-	++	+
Bacillus methylotrophicus B25	-	++	-
Bacillus muralis G25-68	-	+	+
Bacillus mycoides ATCC 6462	-	++	-
Bacillus oceanisediminis 2691	-	+	-
Bacillus paralicheniformis 14DA11	+	++	-
Bacillus pseudofirmus OF4	-	++	+
Bacillus pseudomycoides 219298	-	+	-
Bacillus pumilus B6033	-	++	-
Bacillus safensis KCTC 12796BP L056	-	++	-
Bacillus siamensis SCSIO 05746	-	++	-
Bacillus simplex DSM 1321	-	++	-
Bacillus smithii DSM 4216	-	++	-
Bacillus sonorensis SRCM101395	+	++	-

Bacillus sp. 1NLA3E	-	+	-
Bacillus sp. 1s-1	+	++	-
Bacillus sp. 275	-	++	-
Bacillus sp. ABP14	+	+	-
Bacillus sp. FDAARGOS_235 FDAARGOS_235	+	++	+
Bacillus stratosphericus LAMA 585	-	++	-
Bacillus subtilis ATCC 13952	-	++	-
Bacillus subtilis BEST7613	+	++++	-
Bacillus subtilis spizizenii W23	+	++	-
Bacillus subtilis subtilis BS155	+ *	++	-
Bacillus subtilis subtilis delta6	-	++	-
Bacillus thuringiensis 97-27	+	+	-
Bacillus thuringiensis Bt185	+	++	-
Bacillus thuringiensis HD-771	+	++	-
Bacillus thuringiensis MYBT18246	+	+++	-
Bacillus thuringiensis YBT-1518	+	++++	-
Bacillus toyonensis BCT-7112	+	++	-
Bacillus vallismortis DSM 11031	+	+++	-
Bacillus vallismortis NBIF-001	-	++	-
Bacillus weihaiensis Alg07	-	+	-
Bacillus weihenstephanensis KBAB4	-	++	-
Bacillus xiamenensis VV3	-	+	-
Brevibacillus agri DSM 6348	-	+	-
Brevibacillus brevis NBRC 100599	-	+	-
Brevibacillus formosus NF2	-	+	-

Brevibacillus laterosporus DSM 25	-	+	-
Brochothrix thermosphacta TMW 2.1564	-	-	+
Carboxydocella thermautotrophica 041	-	++	-
Carnobacterium divergens TMW 2.1579	-	-	++
Carnobacterium inhibens subsp. gilichinskyi WN1359	-	-	+
Carnobacterium sp. 17-4	-	-	+
Cellulosilyticum lentocellum RHM5, DSM 5427	-	+	-
Christensenella massiliensis Marseille-P2438	-	-	-
Clostridioides difficile DSM 27640	-	+	-
Clostridium aceticum, DSM 1496	-	++	-
Clostridium acetobutylicum DSM 1731	-	+	-
Clostridium argentinense 89G	-	-	-
Clostridium autoethanogenum DSM 10061	-	-	-
Clostridium baratii CDC51267	-	-	-
Clostridium beijerinckii 59B	-	++	-
Clostridium beijerinckii Br21	-	+	-
Clostridium bornimense M2/40	-	+	-
Clostridium botulinum 111	-	+	-
Clostridium butyricum KNU-L09	-	+	-
Clostridium carboxidivorans P7	-	+	-
Clostridium cellulovorans 743B, ATCC 35296	-	++	-
Clostridium chauvoei 12S0467	-	-	-
Clostridium cochlearium NCTC 13027	-	-	-
Clostridium estertheticum estertheticum DSM 8809	-	+	-

Clostridium formicaceticum DSM 92	-	+	-
Clostridium haemolyticum NCTC 9693	-	+	-
Clostridium isatidis DSM 15098	-	-	-
Clostridium kluyveri DSM 555	-	+	-
Clostridium ljungdahlii PETC, DSM 13528	-	-	-
Clostridium novyi 150557	-	-	-
Clostridium pasteurianum M150B	-	+	-
Clostridium perfringens CP15	-	+	-
Clostridium saccharoperbutylacetonicum N1-4(HMT)	-	+	-
Clostridium scatologenes ATCC 25775	-	+	-
Clostridium septicum DSM 7534	-	-	-
Clostridium sporogenes NCIMB 10696	-	+	-
Clostridium taeniosporum 1/k	-	+	-
Clostridium tetani 12124569	-	-	-
Clostridium tyrobutyricum Cirm BIA 2237	-	+	-
Cohnella sp. KCTC 43028	-	+	-
Coprococcus catus GD/7	-	+	-
Dehalobacter sp. CF	-	+	-
Dehalobacterium formicoaceticum DMC	-	+	-
Desulfallas gibsoniae Groll, DSM 7213	-	+	-
Desulfitobacterium hafniense DCB-2	-	+	-
Desulfofarcimen acetoxidans DSM 771	-	+	-
Desulfosporosinus meridiei S10, DSM 13257	-	++	-
Desulfotomaculum ferrireducens GSS09	-	+	-
Enterococcus avium 352	-	-	+

Enterococcus casseliflavus EC20	-	-	+
Enterococcus cecorum SA3	-	-	+
Enterococcus durans FDAARGOS_234	-	-	+
Enterococcus faecalis KB1	-	-	+
Enterococcus faecium ERS2704476	-	-	+
Enterococcus gallinarum FDAARGOS_163	-	-	+
Enterococcus hirae ATCC 9790	-	-	-
Enterococcus hirae R17	-	-	+
Enterococcus mundtii Pe161	-	-	+
Enterococcus rotai LMG 26678	-	-	+
Enterococcus silesiacus LMG 23085	-	-	+
Enterococcus thailandicus a523	-	-	+
Enterococcus wangshanyuanii MN05 Bos grunniens	-	-	+
Erysipelothrix rhusiopathiae ML101	-	+	-
Eubacterium eligens ATCC 27750	-	+	-
Faecalibacterium prausnitzii L2-6	-	+	-
Fictibacillus arsenicus G25-54	-	+	-
Fictibacillus phosphorivorans G25-29	-	+	-
Geobacillus kaustophilus HTA426	+	-	-
Geobacillus kaustophilus NBRC 102445	+	-	-
Geobacillus lituanicus N-3	+	-	-
Geobacillus sp. 12AMOR1	+	-	-
Geobacillus sp. Y4.1MC1	+	-	-
Geobacillus stearothermophilus 10	+	-	-
Geobacillus subterraneus KCTC 3922	+	-	-

Geobacillus thermocatenulatus KCTC 3921	+	-	-
Geobacillus thermodenitrificans NG80-2	+	-	-
Geobacillus thermoleovorans SGAir0734	+	-	-
Gottschalkia acidurici 9a	-	++	-
Halothermothrix orenii H 168	-	+	-
Heliobacterium modesticaldum Ice1	-	+	-
Jeotgalicoccus saudimassiliensis 13MG44_air	-	+	-
Kyrpidia spormannii EA-1	-	++	-
Kyrpidia tusciae T2, DSM 2912	-	++	-
Lachnoclostridium phytofermentans ISDg	-	+	-
Lachnoclostridium saccharolyticum WM1, DSM 2544	-	+	-
Lacticaseibacillus casei ATCC 334	-	-	+
Lacticaseibacillus paracasei 8700:2	-	-	+
Lacticaseibacillus rhamnosus CECT8800	-	-	+
Lactiplantibacillus paraplantarum DSM 10667	-	-	+
Lactiplantibacillus pentosus DSM 20314	-	-	+
Lactiplantibacillus plantarum 16	-	-	+
Lactobacillus acetotolerans NBRC 13120	-	-	-
Lactobacillus acidipiscis ACA-DC 1533	-	-	+
Lactobacillus acidophilus FSI4	-	-	-
Lactobacillus agilis La3	-	-	+
Lactobacillus alimentarius DSM 20249	-	-	-
Lactobacillus amylovorus 30SC	-	-	-
Lactobacillus backii TMW 1.2002	-	-	+

Lactobacillus brevis CD0817	-	-	-
Lactobacillus brevis TMW 1.2108	-	-	+
Lactobacillus buchneri NRRL B-30929	-	-	+
Lactobacillus casei ATCC 334	-	-	+
Lactobacillus coryniformis coryniformis DSM 20001	-	-	+
Lactobacillus curvatus DSM 20019	-	-	-
Lactobacillus delbrueckii JCM 17838	-	-	+
Lactobacillus fermentum B1 28	-	-	+
Lactobacillus gallinarum HFD4	-	-	+
Lactobacillus helveticus CAUH18	-	-	-
Lactobacillus helveticus MB2-1	-	-	+
Lactobacillus kunkeei MP2	-	-	+
Lactobacillus lindneri TMW 1.481	-	-	+
Lactobacillus oris J-1	-	-	+
Lactobacillus parabuchneri FAM21731	-	-	+
Lactobacillus paracasei 8700:2	-	-	+
Lactobacillus paracollinoides TMW 1.1995	-	-	+
Lactobacillus pentosus SLC13	-	-	+
Lactobacillus plantarum 16	-	-	+
Lactobacillus reuteri CSF8	-	-	-
Lactobacillus rhamnosus ASCC 290	-	-	+
Lactobacillus sakei DS4	-	-	-
Lactococcus garvieae 122061	-	-	-
Lactococcus lactis AI06	-	-	-
Lactococcus lactis cremoris 158	-	-	-

Lactococcus lactis lactis 14B4	-	-	-
Lactococcus piscium CMTALT02	-	-	++
Lactococcus raffinolactis WiKim0068	-	-	++
Lentibacillus amyloliquefaciens LAM0015	-	+	-
Leuconostoc carnosum JB16	-	-	+
Leuconostoc citreum EFEL 2700	-	-	+
Leuconostoc garlicum KFRI01	-	-	+
Leuconostoc gelidum JB7	-	-	+
Leuconostoc kimchii NKJ218	-	-	+
Leuconostoc lactis WiKim40	-	-	+
Leuconostoc mesenteroides SRCM103356	-	-	+
Leuconostoc suionicum DSM 20241	-	-	+
Listeria innocua sv. 6a Clip11262	-	-	+
Listeria ivanovii ivanovii NCTC 11007	-	-	+
Listeria monocytogenes NCTC7973	-	-	+
Listeria seeligeri sv. 1/2b SLCC3954	-	-	+
Listeria weihenstephanensis WS 4560	-	-	+
Listeria welshimeri NCTC 11857	-	-	+
Lysinibacillus macroides DSM 54	-	+	-
Lysinibacillus sp. 2017	-	++	+
Lysinibacillus sp. B2A1	-	+	-
Lysinibacillus sphaericus 2362	-	+	-
Lysinibacillus sphaericus LMG 22257	-	+	+
Lysinibacillus varians GY32	-	+	-
Macrococcus canis KM45013	-	+	-

Macrococcus caseolyticus IMD0819	-	+	-
Macrococcus sp. IME1552	-	+	-
Marinilactibacillus sp. 15R	-	+	-
Megamonas hypermegale NCTC 10570	-	+	-
Megasphaera stantonii AJH120	-	+	-
Melissococcus plutonius ATCC 35311	-	-	-
Novibacillus thermophilus SG-1	-	+	-
Oceanobacillus iheyensis CHQ24	-	+	-
Oceanobacillus sp. 143	-	+	-
Oenococcus kitaharae NRIC 0645, DSM 17330	-	-	+
Oenococcus oeni UBOCC-A-315001	-	-	+
Oenococcus sicerae UCMA15228	-	-	+
Paenibacillus beijingensis DSM 24997	-	+	-
Paenibacillus chitinolyticus KCCM 41400	-	++	-
Paenibacillus mucilaginosus 3016	-	++	+
Paenibacillus physcomitrellae XB	-	+	-
Paenibacillus polymyxa M1	-	+	+
Paenibacillus sp. 32O-W	-	+	-
Paenibacillus sp. FSL R7-0273	-	+	+
Paeniclostridium sordellii JGS6382	-	+	-
Paraclostridium benzoelyticum JC272	-	+	-
Parageobacillus genomosp. 1 NUB3621	+	-	-
Parageobacillus thermoglucosidasius C56-YS93	+	+	-
Paraliobacillus sp. X-1125	-	+	-
Pediococcus acidilactici SRCM101189	-	-	+

Pediococcus claussenii TMW 2.54	-	-	+
Pediococcus damnosus TMW 2.1532	-	-	-
Pelosinus fermentans JBW45	-	++	-
Pelotomaculum thermopropionicum SI	-	+	-
Peptoclostridium acidaminophilum al-2	-	+	-
Peptoclostridium difficile FDAARGOS_267	-	+	-
Planococcus kocurii JCM 2569	-	++	-
Planococcus versutus L10.15	-	++	-
Ruegeria sp. NKC1-1	-	+	-
Rummeliibacillus stabekisii PP9	+	-	-
Salinicoccus halodurans H3B36	-	+	-
Selenomonas sp. F0592	-	+	-
Solibacillus silvestris DSM 12223	-	+	+
Solibacillus sp. R5-41	-	+	-
Sporosarcina pasteurii BNCC 337394	-	+	+
Sporosarcina sp. PTS2304	-	+	-
Staphylococcus agnetis 908	-	+	-
Staphylococcus argenteus BN75	+	-	-
Staphylococcus aureus aureus MSHR1132	+	-	-
Staphylococcus aureus aureus USA300_FPR3757	+	-	-
Staphylococcus capitis AYP1020	+	-	-
Staphylococcus carnosus TM300	-	+	-
Staphylococcus cohnii SNUDS-2	+	-	-
Staphylococcus condimenti DSM 11674	-	+	-
Staphylococcus epidermidis RP62A	+	-	-

Staphylococcus equorum KS1039	+	-	-
Staphylococcus felis ATCC 49168	-	+	-
Staphylococcus haemolyticus JCSC1435	+	-	-
Staphylococcus hominis hominis K1	+	-	-
Staphylococcus hyicus ATCC 11249	-	+	-
Staphylococcus lugdunensis C_33	+	-	-
Staphylococcus lutrae ATCC 700373	-	+	-
Staphylococcus muscae NCTC 13833	-	+	-
Staphylococcus nepalensis JS1	+	-	-
Staphylococcus pasteuri SP1	+	-	-
Staphylococcus pettenkoferi FDAARGOS_288	-	+	-
Staphylococcus piscifermentans NCTC 13836	-	+	-
Staphylococcus pseudintermedius ED99	-	+	-
Staphylococcus saprophyticus 883	+	-	-
Staphylococcus schleiferi 1360-13	-	+	-
Staphylococcus sciuri SNUSD-18	(+)	+	-
Staphylococcus simiae NCTC 13838	+	-	-
Staphylococcus simulans FDAARGOS_124	-	+	-
Staphylococcus stepanovicii NCTC 13839	-	+	-
Staphylococcus succinus 14BME20	+	-	-
Staphylococcus warneri SG1	+	-	-
Staphylococcus xylosus SMQ121	+	-	-
Streptococcus acidominimus NCTC 11291	-	+	-
Streptococcus agalactiae ILRI005	-	+	-
Streptococcus anginosus C1051	-	-	-

Streptococcus australis NCTC 13166	-	+	-
Streptococcus constellatus pharyngis C1050	-	-	-
Streptococcus cristatus NCTC 13807	-	+	-
Streptococcus dysgalactiae equisimilis 167	-	+	-
Streptococcus equi equi 4047	-	+	-
Streptococcus equi zooepidemicus ATCC 35246	-	-	-
Streptococcus equinus FDAARGOS_251	-	+	+
Streptococcus equinus NCTC11436	-	+	-
Streptococcus gallolyticus UCN34	-	+	-
Streptococcus gordonii Challis CH1	-	+	-
Streptococcus halotolerans HTS9	-	-	-
Streptococcus himalayensis HTS2	-	+	-
Streptococcus infantarius ICDDRB-NRC-S5	-	+	-
Streptococcus infantarius infantarius CJ18	-	+	+
Streptococcus infantis SK1302	-	+	-
Streptococcus iniae 89353	-	+	-
Streptococcus intermedius B196	-	-	-
Streptococcus lutetiensis 033	-	+	-
Streptococcus macedonicus ACA-DC 198	-	+	-
Streptococcus marmotae HTS5	-	+	-
Streptococcus merionis NCTC 13788	-	-	-
Streptococcus mitis SVGS_061	-	+	-
Streptococcus mutans LAR01	-	+	+
Streptococcus oligofermentans AS 1.3089	-	+	-
Streptococcus oralis tigurinus osk_001	-	+	-

Streptococcus pantholopis TA 26	-	+	-
Streptococcus parasanguinis FW213	-	+	-
Streptococcus parauberis NCFD 2020	-	+	-
Streptococcus pasteurianus ATCC 43144	-	+	-
Streptococcus pluranimalium TH11417	-	+	-
Streptococcus pneumoniae NCTC 7466	-	+	-
Streptococcus porcinus sv. group v NCTC 10999	-	+	-
Streptococcus pseudopneumoniae IS7493	-	+	-
Streptococcus pyogenes M28PF1	-	+	-
Streptococcus respiraculi HTS25	-	+	-
Streptococcus ruminantium GUT-187	-	+	-
Streptococcus salivarius 57.I	-	+	+
Streptococcus sanguinis SK36	-	+	-
Streptococcus sobrinus NIDR 6715-7	-	+	-
Streptococcus sp. FDAARGOS_192	-	+	+
Streptococcus sp. I-P16	-	+	-
Streptococcus suis DN13	-	+	-
Streptococcus thermophilus CS8	-	-	-
Streptococcus thermophilus LMG 18311	-	+	+
Streptococcus thermophilus M17PTZA496	-	+	++
Streptococcus tigurinus 2426	-	+	-
Streptococcus troglodytae TKU 31	-	+	-
Streptococcus uberis 0140J	-	+	-
Sulfobacillus acidophilus NAL, DSM 10332	+	+	-
Sulfobacillus acidophilus TPY	+	+	-

Syntrophobotulus glycolicus FlGlyR, DSM 8271	-	+	-
Syntrophomonas wolfei Goettingen, DSM 2245B	-	+	-
Terribacillus aidingensis MP602	-	+	-
Tetragenococcus halophilus NBRC 12172	-	-	+
Thermaerobacter marianensis 7p75a, DSM 12885	+	-	-
Thermincola potens JR	-	++	-
Vagococcus sp. HDW17A	-	-	+
Veillonella atypica OK5	-	++	-
Virgibacillus dokdonensis 21D	-	++	-
Virgibacillus halodenitrificans Bac324	-	+	-
Virgibacillus sp. 6R	-	+	-
Virgibacillus sp. Bac330	-	++	-
Virgibacillus sp. Bac332	+	+	-
Weissella ceti WS105	-	-	+
Weissella cibaria CMS2	-	-	+
Weissella confusa VTT E-90392	-	-	+
Weissella paramesenteroides FDAARGOS_414	-	-	+
Weissella soli KACC 11848	-	-	+
Weissella viridescens MFPC16A2805	-	-	+

The presence of the proteins were inferred based on the following PFam domains search from finished bacterial genomes in the Integrated Microbial Genomes & Microbiomes (IGM/G) database: MpsAB (Pfam00361 and PFam10070 respectively), prokaryotic type-carbonic anhydrase (PFam00484) and eukaryotic-type CA (PFam00194). Other PFam domains such as PFam08936 for carboxysome shell carbonic anhydrase (CsoSCA), PFam18484 for cadmium CA repeat and PFam10563

for putative CA-like domain were also searched within the above Firmicutes species but these domains were not found. The terms β-CA for PFam00484 and α-CA for PFam00194 are used in the table in order to more adequately describe the evolutionary history of these enzymes. The symbol +/- indicates the presence or absence of the protein domains. (+) In *S. sciuri* SNUSD-18, MpsA and MpsB appears to be truncated. * contains 2-3 smaller MpsB domains

Supplementary Table 2: Bacterial strains used in this study

Strain/plasmid	Description	Reference
E. coli		
E. coli EDCM636 (*E.coli*Δ*can)*	*E. coli* MG1655 (Δ*canA1*:: *FLK2(lacZ,kan),* λ^-*,* Δ*fnr-267,rph-1)* replacement of *can* by kanamycin resistance marker	(2)
E. coli EDCM636 *can*Sc	*E.coli* EDCM636 (pRB473-*can*Sc) carrying CA from *S. carnosus* TM300	This study
E. coli EDCM636 pRB473	*E.coli* EDCM636 carrying empty plasmid (pRB473)	This study
E. coli EDCM636 *can*Sp	*E.coli* EDCM636 (pCtuf-*can*Sp) carrying CA from S. *pseudintermedius* ED99	This study
E. coli EDCM636 pCtuf	*E.coli* EDCM636 carrying empty plasmid (pCtuf)	This study
E. coli EDCM636 GST-CASc	*E.coli* EDCM636 (pGEX- GST-CASc) complemented with CA from *S. carnosus* TM300 fused with GST tag)	This study
E. coli EDCM636 (pGEX-GST)	*E.coli* EDCM636 carrying empty plasmid with GST tag (pGEX-GST)	This study
S. carnosus		
S. carnosus TM300	Food grade staphylococcal strain lacking genes involved in pathogenicity	(3)
S. carnosus TM300Δ*can*	*S. carnosus* TM300Δ*can* (markerless deletion of Sca_1457)	This study

S. carnosus TM300Δ*can* (pRB473-*can*Sc)	*S. carnosus* TM300Δ*can* complemented with its own CA	This study
S. carnosus TM300Δ*can* (pRB473)	*S. carnosus* TM300Δ*can* carrying empty plasmid (pRB473)	This study
S. carnosus TM300Δ*can* (pRB473-*mpsABC*)	*S. carnosus* TM300Δ*can* complemented with *mpsABC* from *S. aureus*	This study
S. carnosusTM300 pTX30 *icaADBC*	*S. carnosus* TM300 transformed with *icaADBC* from *S. epidermidis* RP62A	(4)
S. carnosusTM300 pTX30	*S. carnosus* TM300 transformed with empty plasmid pTX30	This study
S. carnosus TM300Δ*can* pTX30 *icaADBC*	*S. carnosus* TM300Δ*can* TM300 transformed with *icaADBC* from *S. epidermidis* RP62A	This study
S. carnosus TM300Δ*can* pTX30	*S. carnosus* TM300Δ*can* TM300 transformed with empty plasmid pTX30	This study
S. pseudintermedius		
S. pseudintermedius ED99	Clinical isolate of canine pathogen	(5)
S. pseudintermedius ED99Δ*can*	*S. pseudintermedius* ED99 (markerless deletion of SPSE_0869)	This study

S. pseudintermedius ED99Δ*can* (pCtuf-*can*Sp)	*S. pseudintermedius* ED99Δ*can* complemented with its own CA	This study
S. pseudintermedius ED99Δ*can* (pCtuf)	*S. pseudintermedius* ED99Δ*can* carrying empty plasmid (pCtuf)	This study
S. pseudintermedius ED99Δ*can* (pRB473-*mpsABC*)	*S. pseudintermedius* ED99Δ*can* complemented with *mpsABC* from *S. aureus*	This study
S. aureus		
S. aureus RN4220	*S. aureus* NCTC8325-4 derivative, restriction deficient strain for cloning	(6)
S. aureus HG001	*S. aureus* NCTC8325 derivative with repaired *rsbU*	(7)
S. aureus HG001Δ*mpsABC*	*S. aureus* HG001 markerless deletion of *mpsABC* (SAOUHSC_00412, SAOUHSC_00413, SAOUHSC_00414)	(8)
S. aureus HG001Δ*mpsABC* (pRB473- *can*Sc)	*S. aureus* HG001Δ*mpsABC* complemented with CA from *S. carnosus* TM300	This study
S. aureus HG001Δ*mpsABC* (pRB473)	*S. aureus* HG001Δ*mpsABC* carrying empty plasmid (pRB473)	This study
S. aureus HG001Δ*mpsABC* (pCtuf- *can*Sp)	*S. aureus* HG001Δ*mpsABC* complemented with CA from *S. pseudintermedius* ED99	This study

S. aureus HG001Δ*mpsABC* (pCtuf)	*S. aureus* HG001Δ*mpsABC* carrying empty plasmid (pCtuf)	This study
S. aureus HG001 *can*Sc	*S. aureus* HG001 (pRB473- *can*Sc) carrying CA from *S. carnosus* TM300	This study
S. aureus HG001 *can*Sp	*S. aureus* HG001 (pCtuf- *can*Sp) carrying CA from S. *pseudintermedius* ED99	This study
S. aureus HG001Δ*mpsABC*Δ*ica*	*S. aureus* HG001 markerless deletion of both *mpsABC* and *icaADBC* including its transcriptional regulator (SAOUHSC_03001, SAOUHSC_03002, SAOUHSC_03003, SAOUHSC_03004 and SAOUHSC_03005)	This study
S. epidermidis		
S. epidermidis O47	Clinical isolate from patient with orthopedic implant infection	(9)

Supplementary Table 3: Oligonucleotides used in this study

**Primer name**	**Sequence (5'→3')**
Construction of plasmid pRB473- *can*Sc	
pRB SC CA fwd	GTGCGAATTCGAGCTCGGTACCCATGTTCACAGAAAGAACATTTAGGG
pRB473_Sc CA Rev	GCAGGTCGACTCTAGAGGATCCCCGCAAGTGCATCACACATAACTATT
Construction of plasmid pCtuf- *can*Sp	
pCtuf_sp_CA fwd	AGAGTTCGAGGAGGTTTAATACCAATCTTGGGAGGAAG
pCtuf_sp_CA rev	TTAAGTACTTCAGCTAATTAAATGGTGATGAAACAGTC
Construction of *S. carnosus* TM300Δ*can*	
up_TM300CA_F	CGCGCAGATCTGTCGACGATATATGTGCCGCCACAAGAAT
up_TM300CA_R	TTTTTTACACTTCCTGTTTCCTTAAAA
down_TM300CA_F	TAAGGAAACAGGAAGTGTAAAAAACGGCTATGAATTAGCTGCTC
down_TM300CA_R	TGCAGGCATGCAAGCTTGATCACAACTTGAAATGCGTGCT
Construction of *S. pseudintermedius* ED99Δ*can*	

up_ED99CA_F	CGCGCAGATCTGTCGACGATGCACCGAAAGAAAATCAAACA
up_ED99CA_R	TAGACTTCCTCCCAAGATTGGTACAGTG
down_ED99CA_F	TGTACCAATCTTGGGAGGAAGTCTACGCGCTGAATCCTATCAAA
down_ED99CA_R	TGCAGGCATGCAAGCTTGATAAAGAAGCAATCGCAAAAGC
Construction of *S. aureus* HG001Δ*mpsABC*Δ*ica*	
up_ica_F	GTATTGAACCTAAAATAGGTAATC
up_ica_R	TTTTTGTTACTAGTTTGTAATAATTAAC
down_ica_F	TTATTAAGCTATGTTAAAAACACG
down_ica_R	GTTTCAATGGGTTTACAGATG

Supplementary References

1. Chen IA, Chu K, Palaniappan K, Pillay M, Ratner A, Huang J, Huntemann M, Varghese N, White JR, Seshadri R, Smirnova T, Kirton E, Jungbluth SP, Woyke T, Eloe-Fadrosh EA, Ivanova NN, Kyrpides NC. 2019. IMG/M v.5.0: an integrated data management and comparative analysis system for microbial genomes and microbiomes. Nucleic Acids Res 47:D666-D677.
2. Merlin C, Masters M, McAteer S, Coulson A. 2003. Why is carbonic anhydrase essential to *Escherichia coli*? J Bacteriol 185:6415-24.
3. Wagner E, Doskar J, Gotz F. 1998. Physical and genetic map of the genome of *Staphylococcus carnosus* TM300. Microbiology (Reading) 144 (Pt 2):509-17.
4. Gerke C, Kraft A, Sussmuth R, Schweitzer O, Götz F. 1998. Characterization of the N-acetylglucosaminyltransferase activity involved in the biosynthesis of the Staphylococcus epidermidis polysaccharide intercellular adhesin. J Biol Chem 273:18586-93.
5. Ben Zakour NL, Bannoehr J, van den Broek AH, Thoday KL, Fitzgerald JR. 2011. Complete genome sequence of the canine pathogen *Staphylococcus pseudintermedius*. J Bacteriol 193:2363-4.
6. Kreiswirth BN, Lofdahl S, Betley MJ, O'Reilly M, Schlievert PM, Bergdoll MS, Novick RP. 1983. The toxic shock syndrome exotoxin structural gene is not detectably transmitted by a prophage. Nature 305:709–712.
7. Herbert S, Ziebandt AK, Ohlsen K, Schafer T, Hecker M, Albrecht D, Novick R, Götz F. 2010. Repair of global regulators in *Staphylococcus aureus* 8325 and comparative analysis with other clinical isolates. Infect Immun 78:2877-2889.
8. Fan S-H, Ebner P, Reichert S, Hertlein T, Zabel S, Lankapalli AK, Nieselt K, Ohlsen K, Gotz F. 2019. MpsAB is important for *Staphylococcus aureus*

virulence and growth at atmospheric CO2 levels. Nature communications 10:3627.

9. Raue S, Fan SH, Rosenstein R, Zabel S, Luqman A, Nieselt K, Gotz F. 2020. The Genome of *Staphylococcus epidermidis* O47. Front Microbiol 11:2061.
